# Infective endocarditis caused by methicillin-resistant *Staphylococcus aureus *in a young woman after ear piercing: a case report

**DOI:** 10.1186/1752-1947-5-336

**Published:** 2011-08-01

**Authors:** So-Yun Nah, Moon-Hyun Chung, Jae Eun Park, Areum Durey, Mijeong Kim, Jin-Soo Lee

**Affiliations:** 1 Department of Internal Medicine (Infectious Diseases), Inha University Hospital, Incheon City, Joong-gu, Sinheung-dong 3, Republic of Korea

## Abstract

**Introduction:**

Ear piercing is a common practice among Korean adolescents and young women and usually is performed by nonmedical personnel, sometimes under suboptimal hygienic conditions. Consequently, ear piercing has been associated with various infectious complications, including fatal infective endocarditis. We report a case of infective endocarditis that was caused by community-associated methicillin-resistant *Staphylococcus aureus *after ear piercing and that was accompanied by a noticeable facial rash.

**Case presentation:**

A 29-year-old Korean woman underwent ear piercing six days before hospitalization. On admission, she had fever, erythematous maculopapular rashes on her face, signs of generalized emboli, vegetation in her mitral valve, and methicillin-resistant *S. aureus *bacteremia. On the basis of the blood culture results, she was treated with vancomycin in combination with gentamicin. On day six of hospitalization, a rupture of the papillary muscle of her mitral valve developed, and emergency cardiac surgery replacing her mitral valve with a prosthetic valve was performed. After eight weeks of antibiotic therapy, she was treated successfully and discharged without significant sequelae.

**Conclusions:**

Numerable cases of body piercing-related infective endocarditis have been reported, and since ear piercing is commonplace nowadays, the importance of risk recognition cannot be overemphasized. In our report, a patient developed infective endocarditis that was caused by methicillin-resistant *S. aureus *after ear piercing and that was accompanied by an interesting feature, namely facial rash.

## Introduction

Body piercing, especially ear piercing, is popular among teenage girls and young women in Korea. A previous investigation in Korea revealed that 96.5% of female college students underwent ear piercing at a mean age of 19.3 years. Most students underwent piercing at home or at nonmedical facilities such as jewelry stores, beauty salons, or piercing shops [1]. With the increasing number of persons with body piercings, various associated complications have been reported [2,3]. Among the related adverse events, infectious complications are the most frequent, although these are preventable to a certain extent if aseptic methods are used throughout the piercing procedure. There are no established regulations or medical guidelines regarding body piercing in Korea; thus, some of the body piercings here are probably performed under suboptimal hygienic conditions. Localized infections around the pierced sites are relatively common and usually are managed with topical care; as a result, serious sequelae are rare in most healthy persons. These minor infections occasionally are complicated by bacteremia; if *Staphylococcus aureus *is the causative organism, infective endocarditis (IE) is an expected event. Recently, we encountered a case of IE caused by *S. aureus *after ear piercing in a patient who had no underlying cardiac abnormalities. Interestingly, the patient had scattered erythematous maculopapular rashes on her face and *S. aureus *isolated from blood was resistant to methicillin.

## Case presentation

A 29-year-old, previously healthy, non-HIV-infected Korean woman with a headache, blurred vision, and fever was admitted to our hospital. She had no history of intravenous (IV) drug use, vascular catheter use, acupuncture, or any significant medical condition such as congenital heart disease. She had gotten her earlobes pierced at a beauty salon six days before admission.

On admission, her body temperature was 38.4°C, her blood pressure was 108/63 mm Hg, her heart rate was 83 beats per minute, and her respiratory rate was 18 breaths per minute. The puncture sites on both earlobes were tender without signs of inflammation. Various skin lesions were noted: nonpruritic maculopapular rashes on her face, multiple Janeway lesions and pustules on her palms and soles, scattered pustules and microabscesses on her trunk and extremities, and subungual splinter hemorrhages on her fingers. Subconjunctival hemorrhage was also observed; a slit-lamp examination revealed multiple Roth spot-like retinal hemorrhages. Her lung sounds were clear, and no cardiac murmur was audible. Bilateral costovertebral angle tenderness was noted. No remarkable neurological abnormalities, except for blurred vision, were present. Laboratory tests revealed microscopic hematuria and the following values: white blood cell (WBC) count of 7170 cells/μL, platelet count of 60,000 cells/μL, C-reactive protein level of 260 mg/L, and an erythrocyte sedimentation rate of 10 mm/hour. A cerebrospinal fluid (CSF) analysis revealed red blood cell and WBC counts of 100 and 520 cells/μL (71% neutrophils and 2% lymphocytes), respectively; a CSF protein level of 84 mg/dL; and a glucose level of 61 mg/dL. An electrocardiogram revealed no abnormalities except for sinus tachycardia and right axis deviation. A chest radiography did not reveal any specific abnormality. A transthoracic echocardiography performed on day two of hospitalization revealed vegetation attached to the anterior chordae of her mitral valve; no intracardiac thrombi or structural cardiac abnormalities were observed. Brain magnetic resonance imaging revealed multiple embolic infarcts and leptomeningeal enhancement in both hemispheres of her cerebrum and cerebellum. An abdominal computed tomography (CT) scan revealed hypoattenuated lesions in her spleen and kidneys, suggesting the presence of multiple embolic infarcts with abscesses. *S*. *aureus *isolated from three sets of blood cultures was resistant to penicillin and oxacillin but was susceptible to gentamicin, clindamycin, erythromycin, ciprofloxacin, and trimethoprim-sulfamethoxazole. On the basis of the blood culture results, initial therapy with nafcillin and ceftriaxone was substituted with vancomycin in combination with gentamicin. On day eight of hospitalization, gentamicin treatment was discontinued.

On day six of hospitalization, our patient developed dyspnea and hypotension; a transthoracic echocardiography revealed a rupture of the papillary muscle of her mitral valve. An intra-aortic balloon pump was implanted, after which emergency cardiac surgery confirmed vegetation in the ventricular side of her P3 scallop and a rupture of her mitral valve chordae. Her entire mitral valve, including the base of her medial papillary muscle, was removed and replaced with a prosthetic valve. After eight weeks of vancomycin therapy, our patient was discharged without significant sequelae. Her facial rashes disappeared completely. In a transesophageal echocardiography performed before discharge, her prosthetic mitral valve was found to be functioning well; follow-up brain and abdominal CT scans indicated an improvement in her condition.

## Discussion

Serious infections associated with body piercing are rare; however, there has been a rise in the incidence of IE resulting from piercing of various body parts [4]. Out of the 22 cases of body piercing-related IE summarized by Armstrong and colleagues [4], three reports that were tattoo-related or lacked detailed information were excluded from our literature survey. A total of 23 cases, including three cases [5-7] and the present case, revealed that the most frequently identified causative organism was *S. aureus *(11 cases) followed by *Streptococcus *species (five cases), *Staphylococcus epidermidis *(two cases), and *Haemophilus aphrophilus*, *H. parainfluenzae*, and *Neisseria mucosa *(one case each); and blood cultures yielded negative results in two cases. In regard to the antibiotic susceptibility of *S. aureus*, nine cases involved infection with methicillin-susceptible and two with methicillin-resistant *S. aureus *(MRSA). MRSA infection in persons not exposed to hospital or health-care settings (that is, community-associated MRSA, or CA-MRSA) is increasingly being reported in Korea and is mainly isolated in the form of skin and soft-tissue infections or ear infections [8]. With the community-wide dissemination of MRSA, an increase in the incidence of endocarditis caused by CA-MRSA is expected. However, this condition is still uncommon and occurs primarily in IV drug users or individuals with soft-tissue infections [9]; there was only one case report of CA-MRSA-induced IE in Korea until 2009 [10]. Despite its rare occurrence, IE caused by CA-MRSA has been found in two out of 11 patients with body piercing-related *S. aureus *endocarditis; thus, the present case suggests that body piercing may be a risk factor for the development of CA-MRSA infection. Body piercing has several features in common with IV drug use: the use of sharp objects by nonmedical persons, close physical contact, the high risk of minor skin infections, and, possibly, self-management with antiseptics or antibiotics.

In our patient, we noted a characteristic facial rash, which has never been reported in IE cases associated with ear piercing or piercing of other body parts. Also, there was an interesting similarity between the present case and a case reported by us in 2006 [1]; both patients contracted *S. aureus *endocarditis of the mitral valve after having their ears pierced and developed an erythematous maculopapular rash on the face in addition to signs of generalized emboli, and the rash disappeared after successful treatment of IE with antibiotics in both cases.

Although the pathogenesis of cutaneous stigmata associated with IE, such as Osler nodes or Janeway lesions, has been debated, it is now clear that septic emboli are mainly involved in the development of these stigmata [11]. This explains the manifestation of stigmata at sites remote from the heart, usually the terminal phalanges of the fingers and toes and the soles or the palms. On the other hand, the rash in our cases developed in the face exclusively, suggesting the possibility of a mechanism different from that of cutaneous stigmata due to septic embolism (for example, lymphatic spread).

## Conclusions

Among 23 cases of piercing-related IE, only the two cases reported by us mentioned a facial rash; therefore, we assumed that left-sided IE caused by *S. aureus *after ear piercing led to facial rash development. Besides developing septic emboli, our patients developed diffusely scattered, minute erythematous maculopapules, exclusively on the face. The known cause of facial rash after ear piercing is an allergic reaction or localized bacterial infections around the pierced site. Localized diffuse erythema, mainly around a pierced site, may be noted in staphylococcal toxic shock syndrome [2,3]. No case of scattered maculopapular rashes on the whole face has been reported, even among patients who developed *S. aureus *bacteremia after ear piercing [12,13]. We assumed that the underlying mechanism differed from that of septic embolism and that *S. aureus *spread possibly via lymphatics. Regardless of the precise mechanism of facial rash development, the presence of a facial rash in a person with sepsis or septic embolism (or both) after ear piercing suggests the possibility of left-sided valve IE caused by *S*. *aureus*, even if echocardiography performed at an early stage does not reveal valvular abnormalities or vegetation.

## Abbreviations

CA-MRSA: community-associated methicillin-resistant *Staphylococcus aureus*; CSF: cerebrospinal fluid; CT: computed tomography; IE: infective endocarditis; IV: intravenous; MRSA: methicillin-resistant *Staphylococcus aureus*; WBC: white blood cell.

## Consent

Written informed consent was obtained from the patient for publication of this case report and any accompanying images. A copy of the written consent is available for review by the Editor-in-Chief of this journal.

## Competing interests

The authors declare that they have no competing interests.

## Authors' contributions

S-YN was primarily responsible for drafting, submitting, and revising the manuscript and conducting the literature search. M-HC and J-SL critically revised the manuscript for important intellectual content and gave final approval of the version to be published. JEP, AD, and MK assisted in the patient management and literature review. All authors read and approved the final manuscript.
